# Simulation modeling of the long-term evolution of local malaria transmission and acquired immunity in the context of urban growth and urban-rural travel

**DOI:** 10.1186/1475-2875-9-S2-P47

**Published:** 2010-10-20

**Authors:** José G Siri, Zachary Brown, Martin Spielauer

**Affiliations:** 1 Health and Global Change Project, International Institute for Applied Systems Analysis, Laxenburg, Austria, A-2361; 2 Nicholas School of the Environment, Duke University, Durham, NC 27708, USA; 3 Modeling Division, Statistics Canada, Ottawa, Canada K1A0T6

## 

Malaria occurrence is lower in urban versus rural areas of sub-Saharan Africa for a variety of reasons, including limitation of suitable mosquito habitat, generally improved housing standards and access to prevention and treatment, and a relative decrease in the ratio of vectors to humans. Nonetheless, empirical observation confirms that malaria cases, whether locally transmitted or imported, are frequently observed in cities in endemic areas. Theoretical considerations imply that local transmission will decrease as a city grows, transitioning from a relatively malaria-permissive state to one that encourages reduction or extinction. Using a simple deterministic systems dynamic model based on traditional malaria models, we simulate scenarios for the evolution of local transmission rates and acquired immunity in a homogenous urban area with respect to population size, growth rate, and level of suppression of mosquito breeding. In particular, we consider how the timing and extent of decreases in local transmission are mitigated by travel by city dwellers to endemic rural areas and their resulting exposure to infected mosquitoes. We explore the generalizability and sensitivity to assumptions of our results using microsimulation and analytic methods to account for various aspects of environmental (urban/rural) and demographic heterogeneity. The results of these simulation models should inform projections for long-term urban malaria trends, and have implications for the targeting of malaria prevention efforts in urban and peri-urban areas of sub-Saharan Africa.

